# Human Exposure to Early Morning *Anopheles funestus* Biting Behavior and Personal Protection Provided by Long-Lasting Insecticidal Nets

**DOI:** 10.1371/journal.pone.0104967

**Published:** 2014-08-12

**Authors:** Nicolas Moiroux, Georgia B. Damien, Marc Egrot, Armel Djenontin, Fabrice Chandre, Vincent Corbel, Gerry F. Killeen, Cédric Pennetier

**Affiliations:** 1 MIVEGEC (IRD 224-CNRS 5290-UM1-UM2), Institut de Recherche pour le Développement (IRD), Cotonou, Bénin; 2 MIVEGEC (IRD 224-CNRS 5290-UM1-UM2), Institut de Recherche pour le Développement (IRD), Montpellier, France; 3 Centre de Recherche en Entomologie de Cotonou (CREC), Ministère de la Santé, Cotonou, Bénin; 4 Faculté des Sciences et Techniques, Université d’Abomey Calavi, Cotonou, Bénin; 5 Department of Entomology, Kasetsart University, Bangkok, Thailand; 6 Environmental Health and Ecological Sciences Thematic Group, Ifakara Health Institute, Ifakara, Tanzania; 7 Vector Biology Department, Liverpool School of Tropical Medicine, Liverpool, United Kingdom; University of Crete, Greece

## Abstract

A shift towards early morning biting behavior of the major malaria vector *Anopheles funestus* have been observed in two villages in south Benin following distribution of long-lasting insecticidal nets (LLINs), but the impact of these changes on the personal protection efficacy of LLINs was not evaluated. Data from human and *An. funestus* behavioral surveys were used to measure the human exposure to *An. funestus* bites through previously described mathematical models. We estimated the personal protection efficacy provided by LLINs and the proportions of exposure to bite occurring indoors and/or in the early morning. Average personal protection provided by using of LLIN was high (≥80% of the total exposure to bite), but for LLIN users, a large part of remaining exposure occurred outdoors (45.1% in Tokoli-V and 68.7% in Lokohoué) and/or in the early morning (38.5% in Tokoli-V and 69.4% in Lokohoué). This study highlights the crucial role of LLIN use and the possible need to develop new vector control strategies targeting malaria vectors with outdoor and early morning biting behavior. This multidisciplinary approach that supplements entomology with social science and mathematical modeling illustrates just how important it is to assess where and when humans are actually exposed to malaria vectors before vector control program managers, policy-makers and funders conclude what entomological observations imply.

## Introduction

Recent evidence suggests that malaria vectors may avoid contact with long lasting insecticidal nets (LLINs) by feeding outdoors [1], at times when people do not use them in the early evening [2], [3], and/or early morning [4]. Moiroux and colleagues [4] provide evidence for a shift in malaria vector *Anopheles funestus* biting behavior following an LLIN distribution program with universal coverage targets in two villages in southern Benin, Tokoli-Vidjinnagnimon (Tokoli-V) and Lokohoué. Following LLIN distribution, the peak of biting activity exhibited by *An. funestus* was delayed, even resulting in diurnal biting behavior of a large proportion of the vector population in Lokohoué. Furthermore, the proportion of vectors collected outdoors increased in Tokoli-V. However, human landing catches are not sufficient in themselves to survey patterns of normal human exposure to mosquito bites. Indeed, the timing of human activities, and sleeping behaviors in particular, has a strong modulating effect upon human-mosquito contact and the effectiveness of LLINs that, apart from affecting malaria through a community effect, provide personal protection against bites in specific time and space [2], [5]. Quantifying interactions between mosquitoes and humans is essential to enable meaningful evaluation of personal protection methods. Also, quantifying and characterizing residual malaria transmission [5], [6] will allow targeting with complementary vector control strategies.

In this study, we investigated the interactions between mosquitoes and humans [6] in relation to LLINs use in the same two villages in southern Benin where changes in *An. funestus* biting behavior have recently been observed [4].

## Methods

We carried out mosquito collection in the villages of Tokoli-Vidjinnagnimon (Tokoli-V) (6°26′57.1″ N, 2°09′36.6″ E) and Lokohoué (6°24′24.2″ N, 2°10′32.1″ E) to study the impact of mass distributions of LLINs with universal coverage on the biting behavior of *A. funestus* [4]. Mosquitoes were collected in April 2011 during six nights in four sites per village, both indoors and outdoors, in the act of biting human volunteers [7] between 23∶00 and 09∶00 [4]. All 1284 mosquito specimens [4] that were classified as members of the Funestus group by morphology [8], [9] were subsequently confirmed to be *An. funestus* Giles by species-specific polymerase chain reaction [10] and all data and analyses presented after refer only to unambiguously identified specimens of this important vector species.

In order to obtain appropriate data regarding relevant human behaviors, we surveyed 289 and 252 individuals living in 100 and 114 randomly selected households in Tokoli-V and Lokohoué, respectively, in March 2013 (dry season). According to an exhaustive census carried out by our team in 2007 [11], these samples represented 86% and 98% of the overall population of Tokoli-V and Lokohoué, respectively. We asked the head of the household the time at which each person who usually leave in the household (1) entered and exited his own house the night preceding the survey and (2) the time each LLIN user entered and exited his sleeping space the night preceding the survey. Insufficiently precise answers were not used for further analysis (Text S1).

Data from the human and *An. funestus* behavioral surveys were used to measure the human exposure to *An. funestus* bites through previously described mathematical models [2] (Text S1).

The average *true* personal protection of using an LLIN (P*), i.e. the proportion of exposure to all bites that would otherwise occur both indoors and outdoors that is prevented by using of LLIN, as well as the proportion of protected exposure which occurred indoors for LLIN users either accounting for the personal protection provided by net use (π_i,n_) or ignoring it to compare with available estimates for unprotected people (π_i_) [6] were calculated. Exposure when sleeping under an LLIN Permanet 2.0 (i.e. the LLIN distributed by the National Malaria Control Program in southern Benin) was assumed to be reduced by 92% as estimated for *An. coluzzii* in Benin in an area (Malanville) with very low levels of pyrethroid resistance [12].

Moreover, to assess the relative importance of the high level of host searching behavior of *An. funestus* during daylight hours after 6∶00, we also calculated the proportion of exposure occurring after 6∶00 for unprotected people (π_d_) and net users (π_d,n_) (Text S1).

### Ethics statement

The IRD (Institut de Recherche pour le Développement) Ethics Committee and the National Research Ethics Committee of Benin approved the study (CNPERS, reference number IRB00006860). All necessary permits were obtained for the described field studies. Mosquito collections were performed in privately owned houses. No mosquito collection was done without the approval of the head of the village, the owner and occupants of the collection house. Mosquito collectors gave their written informed consent and were treated free of charge for malaria presumed illness throughout the study. The field studies did not involve endangered or protected species.

## Results

**Figure 1:**
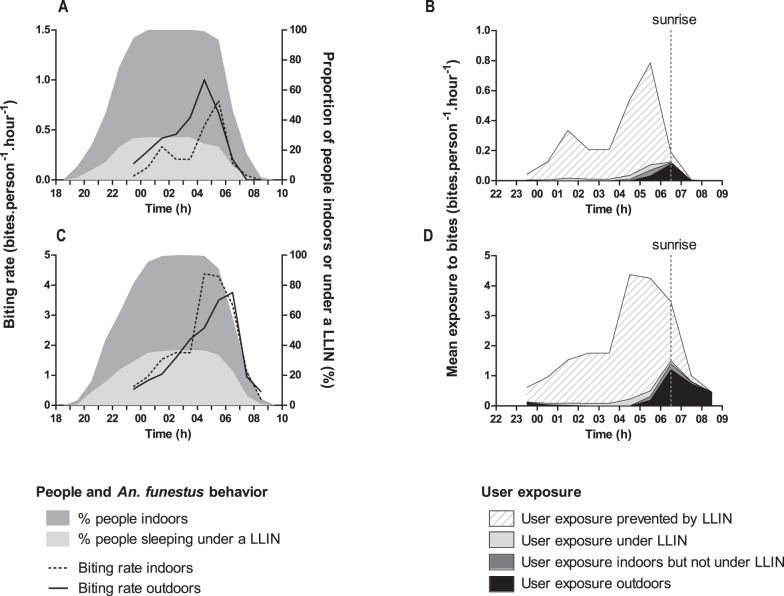
Hourly human and *A. funestus* behavior and hourly exposure to bites of LLIN users in Tokoli-Vidjinnagnimon (A, B) and Lokohoué (C, D).

Figure 1 shows humans and *An. funestus* behavior profiles as well as derived estimates of hourly exposure and prevented exposure to *An. funestus* bite for LLIN users in Tokoli-V and Lokohoué. Only 28.5% and 36.7% of people used LLINs during the human behavioral survey in Tokoli-V and Lokohoué, respectively. Slightly different human behavior profiles between villages were observed: inhabitants of Tokoli-V went both indoors and to bed earlier than Lokohoué inhabitants (Figure 1A and 1C). Most of the total exposure to bites occurred indoors but was largely preventable by using of LLIN (Figure 1B and 1D). The peak of exposure for LLIN users occurred outdoors in and around sunrise from 6∶00 h to 7∶00 h but extended over the following two hours of full daylight in Lokohoué. Raw data are available in Table S1.

Despite the obvious shift to feeding in and around dawn, LLINs were still estimated to provide average ‘true’ personal protection (P*) against 87.1% and 80.3% of exposure to *An. funestus* bites in Tokoli-V and Lokohoué, respectively, because the corresponding proportions of exposure that these LLIN users would have experienced indoors in the absence of nets (π_i_) were 94% and 86.4% (Table 1). However, a large part of the remaining bites that LLIN users are exposed to are estimated to occur outdoors (1−π_i,n_ = 46.1% in Tokoli-V and 68.7% in Lokohoué) and/or after dawn (π_d,n_ = 38.5% in Tokoli-V and 69.4% in Lokohoué).

**Table 1:** *True* average protection efficacy of LLINs against transmission and proportions of indoor and diurnal exposure to bites in Tokoli-Vidjinnagnimon and Lokohoué.

Village	True average LLINpersonal protectionefficacy P* (% [95% CI])	Exposure occurringindoors (% [95% CI])		Exposure occurringafter 6∶00 (% [95% CI])	
		Net users (π_i,n_)	Non-users (π_i_)	Net users (π_d,n_)	Non-users (π_d_)
**Tokoli-V**	87.1 [81.9, 91.2]	54.9 [35.6, 78]	94 [88.7, 97.9]	38.5 [16.2, 59.4]	8.1 [3.4, 13.6]
**Lokohoué**	80.3 [77.1, 83.2]	31.3 [24.4, 39]	86.4 [82.9, 89.5]	69.4 [62.5, 75.7]	24.5 [21.3, 27.8]

LLIN: Long lasting insecticidal net.

## Discussion

This analysis of behavioral interactions between *An. funestus* and humans in two villages of southern Benin showed that the ‘true’ average personal protection of LLINs remained very high (>80%), even when biting activity peaked as net use dropped at dawn [4]. This indicates that LLINs provide, on average, a high level of personal protection even in such a context with clear evidence of increasing diurnal exposure outdoors. While these worrying new vector behaviors are obviously of concern, particularly when viewed in terms of the coverage gap they create [13] in this particular setting, observations of limited impact of LLINs upon disease at the community level [11] appear to be primarily caused by low usage rates (<37% in the present study). Among the reasons for low use of LLINs during the dry season are the higher nocturnal temperatures, the lower biting nuisance [14], and the lack of awareness messaging [15]. Clearly greater efforts are needed to increase the availability of LLINs, to develop more comfortable and convenient bed nets, and to try to increase usage of available nets through awareness campaigns.

To note that the personal protection efficacy P* is an average value for the entire population and some groups of people may be much more exposed. For example, a simple simulation indicates that for very early risers (to bed at 22∶00 and outside the house at 5∶00), personal protection efficacy was 62.5% in Lokohoué, much lower than the average value of 80.3% reported in this study. Moreover, this estimate, as its name implies, refers only to the personal protection provided by using of LLIN and does not give any information about a possible community effect. Indeed, sufficient reductions in mosquito feeding and/or mosquito population may be beneficial to non-user of LLINs in terms of malaria transmission and disease [16].

We found that for LLIN users in Lokohoué, the substantial diurnal behavior of *An. funestus* led to most residual exposure occurring outdoors (68.7%) and/or in the early morning after 6∶00 (69.4%) and in Tokoli-V it approaches 50%. The fact that LLIN user exposure to *An. funestus* bites occurred both indoors and outdoors supports the hypothesis [17], [18] that, even if full universal coverage of LLINs were achieved, both improved indoor control of this highly efficient vector and complementary methods that target vectors outdoors during waking hours or at source [1] will be required to achieve much lower levels of exposure to bites from malaria vectors.

This study has two minor limitations. First, the human behavioral survey was carried out two years after the mosquito collections and human behaviors may have changed between 2011 and 2013. However, we paid great attention to carry out the human behavioral survey during the same period of the year than entomological surveys (end of the long dry season, out of school holydays) to prevent any changes in human behavior in relation to the agricultural or scholar calendars. Moreover in these villages, we have not observed changes (electrification, shift in economic activity…) likely to strongly modify human behaviors. Consequently, the fact that human and mosquito survey do not coincide was less likely to change the results and conclusions. The other issue is that mosquitoes were not collected before 23∶00 and additional biting may have occurred earlier in the evening. Because the majority of people were outdoors before 23∶00 (Figure 1A and 1C), we may have overestimated the proportion of bites occurring indoors and the ‘true’ average personal protection efficacy of LLINs.

To conclude, this study of human-mosquito behavioral interactions highlights the crucial role of LLIN use for personal protection and the possible need to develop new complementary vector control strategies targeting vectors with outdoor and early morning biting behavior. Moreover, this multidisciplinary approach that supplements entomology with social science and mathematical modeling illustrates just how important it is to assess where and when humans are actually exposed to malaria vectors, rather than just where they are caught in surveys of mosquito feeding activity, before vector control program managers, policy-makers and funders conclude what such entomological observations imply.

## Supporting Information

Table S1 Dataset.
(DOC)

Text S1 Questionnaire of the human behavioural survey and formulae used to calculate mean exposure to bite, *true* average personal protection efficacy of LLINs (P*), proportions of indoor exposure to bite (*π_i_* and *π_i,n_*), and proportions of diurnal exposure to bite (*π_d_* and *π_d,n_*).
(DOC)
