# Supplementary material for: Human Exposure to Early Morning Anopheles funestus Biting Behavior and Personal Protection Provided by Long-Lasting Insecticidal Nets
Source: PLoS One. 2014 Aug 12;9(8):e104967. doi: 10.1371/journal.pone.0104967 (PMC4130624; doi:10.1371/journal.pone.0104967)
Supplement: Table S1 — Dataset. (DOC) [file pone.0104967.s001.doc]

|  |  | *An. funestus* collection | | Human behaviour | | | | | |
| --- | --- | --- | --- | --- | --- | --- | --- | --- | --- |
| Lokohouè | hours | nb indoors | nb outdoors | users indoors | users outdoors | users under net | non_users indoors | non-users outdoors | excluded* |
|  | 23-00 | 15 | 13 | 1 | 12 | 53 | 93 | 21 | 72 |
|  | 00-01 | 23 | 20 | 1 | 2 | 63 | 108 | 6 | 72 |
|  | 01-02 | 37 | 25 | 0 | 1 | 65 | 114 | 0 | 72 |
|  | 02-03 | 42 | 38 | 0 | 0 | 66 | 114 | 0 | 72 |
|  | 03-04 | 42 | 53 | 0 | 0 | 66 | 114 | 0 | 72 |
|  | 04-05 | 105 | 62 | 0 | 0 | 73 | 126 | 1 | 52 |
|  | 05-06 | 103 | 84 | 2 | 4 | 68 | 114 | 14 | 50 |
|  | 06-07 | 80 | 90 | 4 | 24 | 46 | 68 | 60 | 50 |
|  | 07-08 | 27 | 23 | 4 | 57 | 13 | 30 | 98 | 50 |
|  | 08-09 | 5 | 11 | 0 | 72 | 2 | 7 | 121 | 50 |
|  |  |  |  |  |  |  |  |  |  |
|  |  | *An. funestus* collection | | Human behaviour | | | | | |
| Tokoli-V | hours | nb indoors | nb outdoors | users indoors | users outdoors | users under net | non_users indoors | non-users outdoors | excluded* |
|  | 23-00 | 1 | 4 | 0 | 1 | 67 | 160 | 11 | 50 |
|  | 00-01 | 3 | 7 | 0 | 0 | 68 | 171 | 0 | 50 |
|  | 01-02 | 8 | 10 | 0 | 0 | 68 | 171 | 0 | 50 |
|  | 02-03 | 5 | 11 | 0 | 0 | 68 | 171 | 0 | 50 |
|  | 03-04 | 5 | 15 | 0 | 0 | 68 | 171 | 0 | 50 |
|  | 04-05 | 13 | 24 | 1 | 0 | 62 | 191 | 2 | 33 |
|  | 05-06 | 19 | 16 | 3 | 3 | 57 | 179 | 14 | 33 |
|  | 06-07 | 4 | 5 | 3 | 31 | 24 | 92 | 106 | 33 |
|  | 07-08 | 1 | 0 | 1 | 50 | 7 | 36 | 162 | 33 |
|  | 08-09 | 0 | 0 | 1 | 57 | 0 | 4 | 194 | 33 |
|  |  |  |  |  |  |  |  |  |  |
| *due to refusal or unsufficiently precise answers to the questionnaire | | | | | |  |  |  |  |
