# Supplementary material for: Human Exposure to Early Morning Anopheles funestus Biting Behavior and Personal Protection Provided by Long-Lasting Insecticidal Nets
Source: PLoS One. 2014 Aug 12;9(8):e104967. doi: 10.1371/journal.pone.0104967 (PMC4130624; doi:10.1371/journal.pone.0104967)
Supplement: Text S1 — Questionnaire of the human behavioural survey and formulae used to calculate mean exposure to bite, true average personal protection efficacy of LLINs (P*), proportions of indoor exposure to bite (πi and πi,n), and proportions of diurnal exposure to bite (πd and πd,n). (DOC) [file pone.0104967.s002.doc]

### Text S1 – Questionnaire of the human behavioural survey and formulae used to calculate mean exposure to bite, *true* average personal protection efficacy of LLINs (*P**), proportions of indoor exposure to bite (*π_i_* and *π_i,n_*), and proportions of diurnal exposure to bite (*π_d_* and *π_d,n_*):

### Questionnaire:

In each randomly selected household, the head was asked to answer the simple following questionnaire for himself/herself and for all the members of the household:

1. To the nearest hour, what time last night did you go indoors for the evening?
2. Did you sleep under a bed net last night?

If yes:

2.1) To the nearest hour, what time did you go to bed last night?

2.2) To the nearest hour, what time did you get out of bed this morning?

1. To the nearest hour, what time did you go outdoors this morning?

The head of the household was asked to answer the same questions for each person who usually leave in the household. Answers given with a precision lower than the nearest hour were excluded from further analyses.

### Formulae used to calculate mean exposure to bite, true average personal protection efficacy of LLINs (P*), proportions of indoor exposure to bite (πi and πi,n), and proportions of diurnal exposure to bite (πd and πd,n):

Data collected from LLIN users during the human behavioural survey were used to calculate the proportions of people declaring being indoors (*I_t_*) and the proportion of people declaring being protected by a LLIN (*S_t_*) for each surveyed hour of the night (*t*), which included 10 hours from 23:00 to 9:00, which were numbered 0, 1, 2 … 9. These data were used to calculate the mean biting rate experienced by an unprotected person and a protected net user (*n*).

The **mean biting rate experienced by a non-user (*B_t_*)** for each surveyed time of the night (t) can be calculated as the proportion of people declaring being indoors (*I_t_*) multiplied by the indoor biting rate at that time (*B_i,t_*) plus the proportion of people declaring being outdoors (*1- I_t_*) multiplied by the outdoor biting rate at that time (*B_o,t_*):

$B_{t}=B_{i,t}I_{t}+B_{o,t}\left(1-I_{t}\right)$ (1)

Then **the mean biting rate (*B*) experienced by a non-user** between 23:00 and 9:00 can be calculated by summing hourly biting rates as follow:

$B_{0\to9}=\sum_{t=0}^{9} B_{t}$ (2)

The **mean biting rate experienced by a LLIN user (*B_n_*)** between 23:00 and 9:00 was calculated by adjusting the indoor biting rate for the protected fraction (*S_t_*) of the population in proportion to the personal protection (*P*) provided by LLINs Permanet 2.0™ (i.e. the LLINs distributed in the villages of the study) [1, 2]:

$B_{n,0\to9}=\sum_{t=0}^{9} B_{n,t}=\sum_{t=0}^{9} \left[B_{i,t}\left(\left(I_{t}-S_{t}\right)+S_{t}\left(1-P\right)\right)+B_{o,t}\left(1-I_{t}\right)\right]$ (3)

The personal protection (*P*) was set to 0.92 according to experimental hut trials of Permanet 2.0™ in Benin in an area (Malanville) with very low levels of pyrethroid resistance [3].

The ***true* average personal protection efficacy of a LLIN (*P**)** against exposure to bites was calculated as the overall reduction of biting rate for LLIN users (compared to non-users) [1] using the following formula:

$P*=1-\left(B_{n,0\to9}/B_{0\to9}\right)$ (4)

The **proportion of bites experienced indoors by a non-user (*π_i_*)** was calculated by comparing the mean biting rates experienced indoors to the overall mean biting rate:

$\pi_{i}=\sum_{t=0}^{9} \left[B_{i,t}I_{t}\right]/B_{0\to9}$ (5)

The **proportion of bites experienced indoors by an LLIN user (*π_i,n_*)** was calculated by comparing the mean biting rates experienced indoors to the overall mean biting rate:

$\pi_{i,n}=\sum_{t=0}^{9} \left[B_{i,t}\left(\left(I_{t}-S_{t}\right)+S_{t}\left(1-P\right)\right)\text{ }\right]/B_{n,0\to9}$ (6)

The **proportion of bites experienced during daylight hours (i.e. between 6:00 and 9:00) by a non-user (*π_d_*)** was calculated by comparing the mean biting rate experienced between 6:00 and 9:00 to the overall mean biting rate (i.e. between 23:00 and 9:00):

$\pi_{d}=B_{6\to9}/B_{0\to9}$ (7)

The **proportion of bites experienced during daylight hours (i.e. between 6:00 and 9:00) by an LLIN user (*π_d,n_*)** during was calculated by comparing the mean biting rate experienced between 6:00 and 9:00 to the overall mean biting rate (i.e. between 23:00 and 9:00):

$\pi_{d,n}=B_{n,6\to9}/B_{n,0\to9}$ (8)

### Confidence intervals for *P*, π_i_, π_i,n_, π_d_,* and *π_d,n_*:

In order to produce Confidence intervals for *P*, π_i_, π_i,n_, π_d_,* and *π_d,n_*, we assumed that counts of *An. funestus* collected indoor and outdoor at time *t* followed a Poisson distribution with means *N_i,t_* and *N_o,t_*, respectively, corresponding to the number of mosquitoes collected during entomological survey. Moreover, we assumed that proportions of LLIN users (1) being outdoors, (2) being indoors and protected with an LLINs, and (3) being indoors and not protected followed a Multinomial distribution with parameter *n* equal to the number of LLIN users and probabilities *p1*, *p2*, and *p3* equal to the measured values of (1-*I_t_*), *S_t_*, and (*I_t_* -*S_t_*), respectively.

For each surveyed time of the night (*t*), we used functions ‘rpois’ and ‘rmultinom’ in the R software to generate 5000 random values *N_i,t,j_*, *N_o,t,j_*, (1-*I_t_*)*_j_*, *S_t,j_* and (*I_t_* -*S_t_*)*_j_*, numbered from *j*=1 to 5000. Random samples of hourly biting rates *B_i,t,j_* and *B_o,t,j_* were obtain by divided *N_i,t,j_* and *N_o,t,j_*, respectively, by the duration *d* of mosquito collections (*d = 24 =* 6 nights x 4 sites per village).

Then we used formulae described above to calculate samples of 5000 random values of *P*, π_i_, π_i,n_, π_d_,* and *π_d,n_*. 2.5 and 97.5 percentiles of the resulting samples were used as boundaries of the 95% confidence intervals.

# References

1. Killeen GF, Kihonda J, Lyimo E, et al. Quantifying behavioural interactions between humans and mosquitoes: evaluating the protective efficacy of insecticidal nets against malaria transmission in rural Tanzania. BMC Infect Dis 2006;6:161

2. Seyoum A, Sikaala CH, Chanda J, et al. Human exposure to anopheline mosquitoes occurs primarily indoors, even for users of insecticide-treated nets in Luangwa Valley, South-east Zambia. Parasit Vectors 2012;5:101

3. Corbel V, Chabi J, Dabire RK, et al. Field efficacy of a new mosaic long-lasting mosquito net (PermaNet 3.0) against pyrethroid-resistant malaria vectors: a multi centre study in Western and Central Africa. Malar J 2010;9:113
